# Left ventricular torsion rate and the relation to right ventricular function in pediatric pulmonary arterial hypertension

**DOI:** 10.1177/2045894018791352

**Published:** 2018-07-13

**Authors:** Melanie J. Dufva, Uyen Truong, Pawan Tiwari, Dunbar D. Ivy, Robin Shandas, Vitaly O. Kheyfets

**Affiliations:** 1 Department of Bioengineering, University of Colorado Denver, USA; 2 Department of Pediatrics, Section of Cardiology, Children's Hospital Colorado, USA

**Keywords:** pulmonary hypertension, torsion mechanics, pediatrics, tagged cardiac magnetic resonance

## Abstract

The right ventricle and left ventricle are physically coupled through the interventricular septum. Therefore, changes in the geometry and mechanics of one ventricle can directly affect the function of the other. In treatment of pediatric pulmonary arterial hypertension, the left ventricle is often overlooked, with clinical focus primarily on improving right ventricular function. Pediatric pulmonary arterial hypertension represents a disease distinct from adult pulmonary arterial hypertension based on etiology and survival rates. We aimed to assess left ventricular torsion rate in pediatric pulmonary arterial hypertension and its role in right ventricular dysfunction. Cardiac magnetic resonance images with tissue tagging were prospectively acquired for 18 pediatric pulmonary arterial hypertension (WHO class I) patients and 17 control subjects with no known cardiopulmonary disease. The pulmonary arterial hypertension cohort underwent cardiac magnetic resonance within 48 hours of clinically indicated right heart catheterization. Using right heart catheterization data, we computed single beat estimation of right ventricular end-systolic elastance (as a measure of right ventricular contractility) and ventricular vascular coupling ratio (end-systolic elastance/arterial afterload). Left ventricular torsion rate was quantified from harmonic phase analysis of tagged cardiac magnetic resonance images. Ventricular and pulmonary pressures and pulmonary vascular resistance were derived from right heart catheterization data. Right ventricular ejection fraction and interventricular septum curvature were derived from cardiac magnetic resonance. Left ventricular torsion rate was significantly reduced in pulmonary arterial hypertension patients compared to control subjects (1.40 ± 0.61° vs. 3.02 ± 1.47°, *P* < 0.001). A decrease in left ventricular torsion rate was significantly correlated with a decrease in right ventricular contractility (end-systolic elastance) (*r* = 0.61, *P* = 0.007), and an increase in right ventricular systolic pressure in pulmonary arterial hypertension kids (*r* = –0.54, *P* = 0.021). In both pulmonary arterial hypertension and control subjects, left ventricular torsion rate correlated with right ventricular ejection fraction (controls *r* = 0.45, *P* = 0.034) (pulmonary arterial hypertension *r* = 0.57, *P* = 0.032). In the pulmonary arterial hypertension group, interventricular septum curvature demonstrated a strong direct relationship with right ventricular systolic pressure (*r* = 0.7, *P* = 0.001) and inversely with left ventricular torsion rate (*r* = –0.57, *P* = 0.013). Left ventricular torsion rate showed a direct relationship with ventricular vascular coupling ratio (*r* = 0.54, *P* = 0.021), and an inverse relationship with mean pulmonary arterial pressure (*r* = –0.60, *P* = 0.008), and pulmonary vascular resistance (*r* = –0.47, *P* = 0.049). We conclude that in pediatric pulmonary arterial hypertension, reduced right ventricular contractility is associated with decreased left ventricular torsion rate.

## Introduction

Pulmonary arterial hypertension (PAH) in children is an incurable and progressive disease, characterized by increasing mean pulmonary arterial pressure (mPAP) and vascular resistance, resulting in right ventricular (RV) hypertrophy, and eventual ventricular failure.^1–3^ In contrast to adult PAH, pediatric PAH etiology is predominantly idiopathic and associated with congenital heart disease^4^ and has been shown to have a worse survival,^5^ with adult studies greatly outnumbering pediatric research. Thus, a study of the progression of PAH in children, separate from adult pathophysiology, is needed.

During PAH progression, the rise in RV pressure causes alteration in the interventricular septal (IVS) geometry,^6,7^ which interferes with normal left ventricular (LV) geometry and mechanics, thus affecting RV–LV interdependent mechanical and structural function.^8–10^ However, the contribution of the left ventricle on RV function is often overlooked in the clinical setting in the management of PAH, in which current therapies primarily focus on RV function improvement.^10^ A study conducted by Fogel et al. on patients with systemic right ventricles observed differences in ventricular strain, strain rate, and ventricular twisting compared to normal healthy subjects, demonstrating that the lack of normal LV function has a significant effect on RV mechanics.^11^ Damiano et al. showed that in an electrically isolated, healthy, dog heart, the left ventricle alone contributed to passive RV contraction by inducing 68% of normal RV systolic pressure and 80% of pulmonary arterial flow.^12^ These studies demonstrate the critical role the left ventricle has in affecting RV systolic function. LV torsion and rotation may reflect the ability of the left ventricle to contract and transfer mechanical energy to the right ventricle, and has been shown to be indicative of LV ejection, filling, and potential energy storage.^13,14^ Therefore, we hypothesize that the LV torsion rate is decreased in our sample of children with PAH, and that this reduction is associated with a decrease in RV function (e.g. contractility and ejection fraction), RV–pulmonary arterial decoupling (measured as the ventricular–vascular coupling ratio), and hemodynamic measures of pulmonary arterial and ventricular afterload.

## Methods

To test our hypothesis, we prospectively recruited PAH patients undergoing clinically indicated right heart catheterization (RHC) to undergo tagged cardiac magnetic resonance imaging (MRI) within 48 hours. Cardiac magnetic resonance (CMR) imaging tissue tagging is the reference standard for evaluating myocardial deformation.^15^ Like CMR, echocardiography can also measure myocardial deformation. However, echo techniques are limited by available acoustic windows and body habitus. Near-field imaging is particularly limited, making the anterior right ventricle often difficult to delineate. CMR approaches are free of these constraints and allow high spatial resolution images without exposure to radiation or sedation in older children.^8^

### Study population

This study was carried out with the approval of the Colorado institutional research board, in accordance with the Declaration of Helsinki. We recruited PAH patients cared for in the pulmonary hypertension program at the Children's Hospital Colorado who were undergoing clinically indicated RHC to undergo CMR within 48 hours. Healthy control subjects were recruited prospectively for CMR alone. The inclusion criteria for this study were any person aged 7–21 years, with mPAP of 25 mmHg or greater established by catheterization or RV pressure of 50% or greater of systemic arterial pressure established by echocardiogram before the age of 18 years for PAH patients. We excluded significant intracardiac shunts defined as a pulmonary:aortic flow of more than 1.2:1. In addition, patients with pulmonary thromboembolic diseases, PAH from left heart disease, veno-occlusive disease, pulmonary capillary hemangiomatosis, or lung disease were excluded from this study. Medical records, including World Health Organization (WHO) functional class, were retrospectively reviewed. Longitudinal disease progression was not considered in this analysis. For any subject, differences in hemodynamic states between sedated RHC and non-sedated CMR were unavoidable. Our institutional protocol calls for RHC under general anesthesia for all children, while our CMR research protocol included older children (>5 years old) without sedation during CMR. Thus, with the youngest subject at age 7 years, no children in either cohort underwent sedation during CMR for this study. Control healthy subjects aged 7–21 years with no known cardiopulmonary disease were recruited by campus advertisement.

### Right heart catheterization

RHC was performed under general anesthesia. Pulmonary vascular resistance (PVR = (mPAP – PCWP)/CO) was taken as a measure of resistive afterload, in which PCWP is the pulmonary capillary wedge pressure, mPAP is mean pulmonary arterial pressure, and CO is cardiac output. The cardiac index was measured using Fick's principle at baseline, with FiO_2_ as close to room air as tolerated.

#### Vascular ventricular coupling ratio

RV end-systolic elastance (E_es_) was used as a measure for RV contractility, which was estimated using the single beat method described by Truong et al.^16^ E_es_ was estimated as the ratio of the difference between maximum pressure and end systolic pressure to stroke volume [(P_max_ – P_es_)/SV]. Arterial elastance (E_a_), which represents arterial afterload, was defined as the ratio of end systolic pressure to stroke volume [P_es_/SV]. Arterial compliance was defined as the ratio of stroke volume to pulse pressure (PP) [SV/PP]. The ventricular vascular coupling ratio (VVCR) was calculated as the ratio of ventricular contractility (E_es_) to arterial afterload (E_a_), with higher VVCR values representing increased coupling competency between the pulmonary artery and right ventricle.^17–19^

### Tagged CMR and post-processing analysis

MRI was performed on a 1.5 T Philips Ingenia scanner (Philips Medical, The Netherlands) with dedicated cardiac receiver coils appropriate for subject size. All subjects were awake during the study. Tagged MRI images are acquired after application of a pre-pulse to nullify signal within defined parallel lines or grids known as ‘tags’ which persist through the cardiac cycle. The low signal intensity lines allow tracking of the myocardium through systole and diastole. This was applied to the horizontal long axis and three short-axis planes (base, mid-papillary, and apical level). Typical image parameters were 6–8 mm slice thickness, repetition time of 3.9–4.7 ms, echo time of 3.7 ms, flip angle 15°, matrix 144 × 256, voxel size of 1.875 × 1.875 × 8 mm, with a tag spacing of 6–8 mm. Images were acquired in short-axis view at the basal, mid, and apical levels with a minimum of 25 phases throughout the cardiac cycle. The short-axis basal slice was defined as a full circumferential view of the LV chamber as landmarked directly inferior to the mitral valve leaflet tips. The short-axis apical slice was defined as a full circumferential view of the chamber inferior to the papillary muscles but superior to the end of the cavity. The mid-chamber slice was defined as the region of the cavity encompassing the entire length of the papillary muscles.

#### LV torsion rate

The LV torsion was calculated over the cardiac cycle using harmonic phase flow analysis (Computer Vision Center, Universitat Autònoma de Barcelona, Barcelona, Spain).^20,21^ The harmonic phase flow method extracts myocardial motion between consecutive tagged CMR image frames by generation of two-dimensional vector fields indicating the position and direction of a point through analysis in the Gabor domain.^22^ LV rotation in degrees was calculated over the cardiac cycle for basal, mid-ventricular, and apical short-axis views. End-systole was defined by aortic valve closure. Torsion was calculated using the equation

(1) $$\text{T}=\frac{\left(\text{BR}-\text{AR})(r_{\text{apex}}+r_{\text{base}}\right)}{2\text{D}}$$

**Fig. 1.**
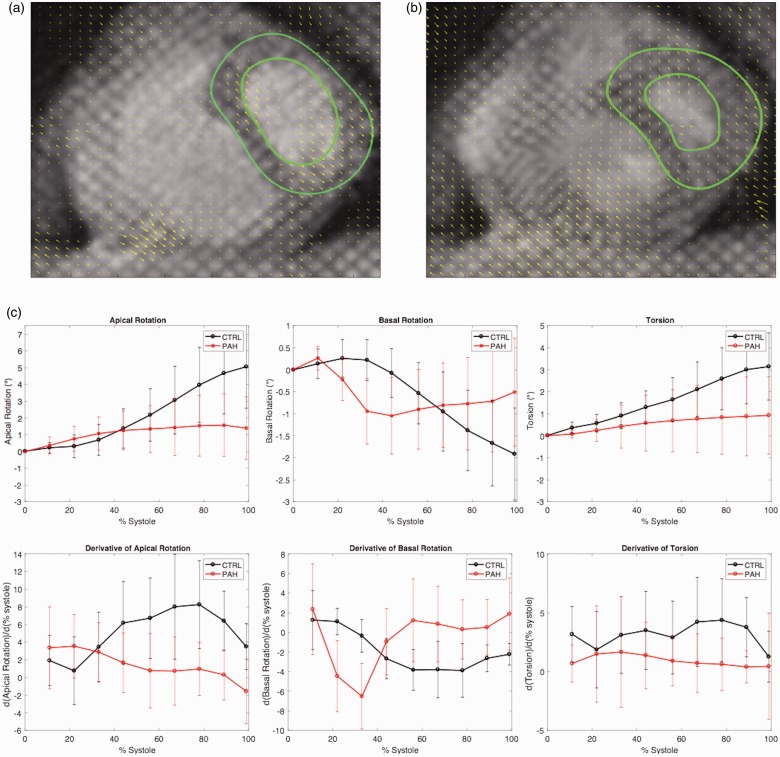
Tagged-magnetic resonance images taken short-axis of the ventricles during (a) the beginning of left ventricular systole and (b) the end of left ventricular systole. Harmonic phase flow processing produces a motion vector field for each frame for subsequent torsion analysis. (c) Apical rotation, basal rotation, torsion and their derivates for control subjects (black) and pulmonary arterial hypertension subjects (red).

In equation (1), BR is the rotation of the base, AR is the rotation of the apex, *r*_apex_ and *r*_base_ are their respective radii, and D is the length between the base and apex in systole.^20^ The radius was measured as half the length of the measured distance between the anterior endocardial wall and the inferior endocardial wall of the left ventricle. Torsion rates were calculated as the change in degrees of torsion per length (cm) over one systolic cycle. Cubic spline interpolation was performed on torsion versus percentage systole with an interval of 0.001. The derivative of the torsion curve (top of Fig. 1c) was taken to derive the torsion rate (bottom of Fig. 1c) with mean values and error bars displayed at intervals of 10%. Planar vector fields were generated for each cardiac frame during systole, as seen in Fig. 1a and b.

#### Septal radius of curvature

The septal radius of curvature (SROC) was utilized as a metric for the extent of septal flattening through analysis of cine CMR. This was measured by fitting a curve at three points along the LV endocardial septal wall, from anterior to posterior, within the basal level in short-axis view, at end-systole. The corresponding SROC was normalized to the distance between the basal and apical planes of the left ventricle. Larger radii represent an increase in septal flattening.

### Statistical analysis

All data analyses were performed using MatLab, in which an alpha of 0.05 represented statistical significance, with a 95% confidence interval that any one sample is representative of the population. The mean and standard deviation of LV torsion rate were calculated for control and PAH patients. An unpaired two-tailed Student's *t*-test was used to compare the mean LV torsion rate between control and PAH subjects. Univariate linear regression was used to assess relationships between torsion rate and mechanical and hemodynamic vascular/ventricular functional parameters (right ventricular ejection fraction (RVEF), mPAP, PVR, VVCR, RV systolic pressure and RV contractility). Reproducibility, and intraobserver and interobserver agreement for harmonic phase flow analysis and the algorithm used have been verified in previous studies;^20–22^ however, we performed our own independent analysis. Intraobserver and interobserver reproducibility of LV rotation and torsion was assessed in 10 randomly selected PAH patients by two blinded researchers (MJD and PT) utilizing intraclass correlation coefficients (ICCs) and Bland–Altman analysis. Intraobserver variability was assessed by comparison of measurements performed by the same observer (MJD) approximately 10 months apart.

## Results

### Patient demographics

Patient demographic details are summarized in Table 1. A total of 36 patients were analyzed, with 18 PAH patients and 17 control subjects. All participants provided written informed consent.

**Table 1.** Patient demographics and hemodynamic characteristics.

Parameter	PAH (*n* = 18)	Control (*n* = 17)	*P* value
Female, *n* (%)	12 (66%)	11 (65%)	0.926
Age, years	13 (9–20)	10 (7–16)	0.077
Weight, kg	50.8 (20.6–67.8)	46.6 (26.4–90.6)	0.976
Height, cm	153.8 (123.7–163)	150 (127–176)	0.518
Body surface area, m^2^	1.53 (0.83–1.76)	1.43 (0.98–1.77)	0.828
Race			
Hispanic or Latino	2 (11%)	2 (12%)	0.953
Caucasian	7 (39%)	9 (53%)	0.613
Asian or Pacific Islander	5 (28%)	2 (12%)	0.249
Other	4 (22%)	1 (0.5%)	0.177
Unknown	0	2 (12%)	0.142
WHO class I	18 (100%)	_	_
mPAP, mmHg	49 (24–58)	–	–
PVR, Pa·s/m^3^	8.50 (2.32–22.76)	–	–
PCWP, mmHg	9 (5–12)	–	–
RVCO, mL/min	5.6 (2.2–11.3)	4.46 (2.51–8.38)	0.054
RVSV, mL	76.2 (26.7–106.8)	63.4 (35.8–97.1)	0.151
RVEF, %	45 (22–54)	58 (47–67)	<0.001
LVEF, %	57 (42–65)	59 (53–66)	0.322

Data are expressed as median values (interquantile range), *n* (%), unless otherwise noted.

PAH: pulmonary arterial hypertension; WHO: World Health Organization; mPAP: mean pulmonary arterial pressure; PVR: pulmonary vascular resistance; RVCO: right ventricular cardiac output; RVSV: right ventricular stroke volume; RVEF: right ventricular ejection fraction; LVEF: left ventricular ejection fraction.

Female patients comprised 66% of the PAH group and 65% of the control group. The median age for the PAH group was 13 (range 9–20) years, compared to 10 (range 7–16) years in the controls. All PAH patients were classified as WHO group I based on medical records. Thirteen of the PAH patients were diagnosed as idiopathic PAH, one patient was diagnosed as PAH due to a connective tissue disease, three had PAH due to congenital heart disease (two with atrial septal defects, one patient with patent ductus arteriosus), and two with hereditary PAH. Median mPAP and PVR for the PAH subjects was 49 (range 24–58) mmHg and 8.50 (range 2.32–22.76) Wood units, respectively. RVEF is significantly lower in the PAH subjects compared to controls. RV CO and RV SV were not significantly different between the cohorts. No significant differences were found in left ventricular ejection fraction (LVEF) between PAH (median 57%, range 42–65%) and control subjects (median 59%, range 53–66%) (*P* = 0.322).

### LV torsion rate and RV function

Apical rotation, basal rotation, and torsion with their derivatives are shown in Fig. 1(c) for PAH (red) and control subjects (black), with means and standard deviations presented at 11%, 22%, 33%, 44%, 56%, 67%, 78%, 89%, and 100% systole. The greatest differences in rotation and torsion between the groups were observed at end-systole. The interobserver analysis revealed good agreement for torsion rate within PAH subjects (ICC 0.84; mean difference 0.14; 95% confidence interval −0.46 to 0.74). Similarly, the intraobserver agreement analysis demonstrated strong agreement (ICC 0.96; mean difference –0.039; 95% confidence interval −0.14 to 0.06) with a separation period of 10 months between analyses. The Bland–Altman plots are displayed in Supplementary Fig. 1.

**Fig. 2.**
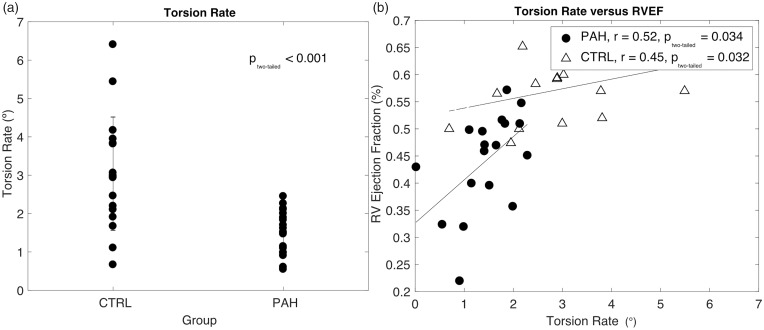
(a) Torsion rates for pulmonary arterial hypertension (PAH) (1.40 ± 0.61°) are significantly reduced from control (3.02 ± 1.47°) patients (*P* < 0.001), and (b) right ventricular (RV) ejection fraction versus torsion rate for PAH (•, *r* = 0.57, *P* = 0.012) and controls (△, *r* = 0.45, *P* = 0.032).

**Fig. 3.**
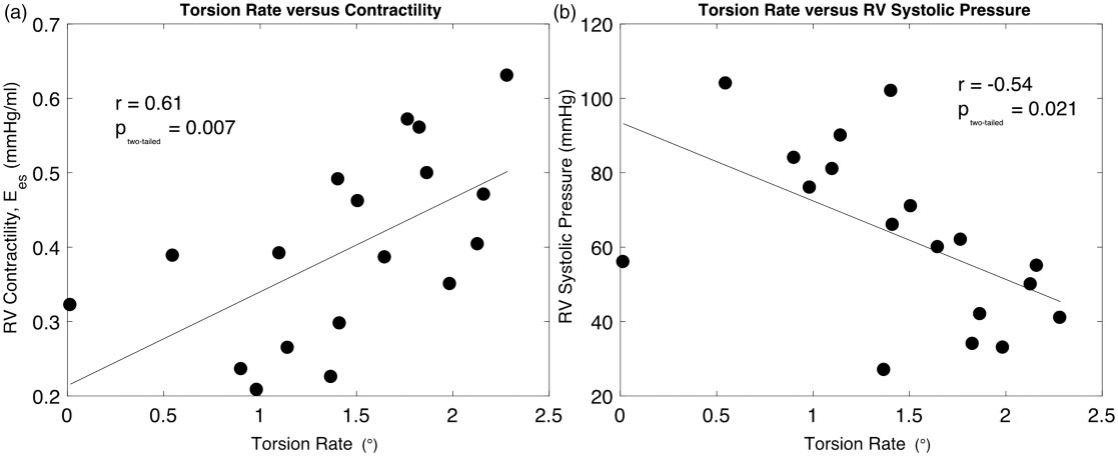
Torsion rates for pulmonary arterial hypertension patients are highly correlated with (a) right ventricular (RV) contractility (*r* = 0.60, *P* = 0.007), and (b) RV systolic pressure (*r* = −0.54, *P* = 0.021).

The LV torsion rate for control and PAH patients is displayed in Fig. 2a. LV torsion rates in the controls are significantly higher than those of PAH patients (3.02 ± 1.47° vs. 1.40 ± 0.61°, *P* < 0.001, respectively). Fig. 2b shows the LV torsion rate positively correlated with RVEF in both controls (*P* = 0.032) and PAH subjects (*P* = 0.034). The graph depicts a steeper slope of RVEF values in response to a change in LV torsion rate in the PAH cohort compared to the controls. Unlike the PAH group, in the control subjects, the torsion rate did not correlate with RVCO or RVSV. The LV torsion rate also demonstrated a positive relationship with E_es_ (RV contractility) (*P* = 0.007), but an inverse relationship with RV systolic pressure (*P* = 0.021), as shown in Fig. 3a and b. The LV torsion rate did not demonstrate a significant relationship with LVEF in control (*r* = 0.03, *P* = 0.909) or PAH subjects (*r* = 0.42, *P* = 0.073).

### LV torsion rate and SROC

**Fig. 4.**
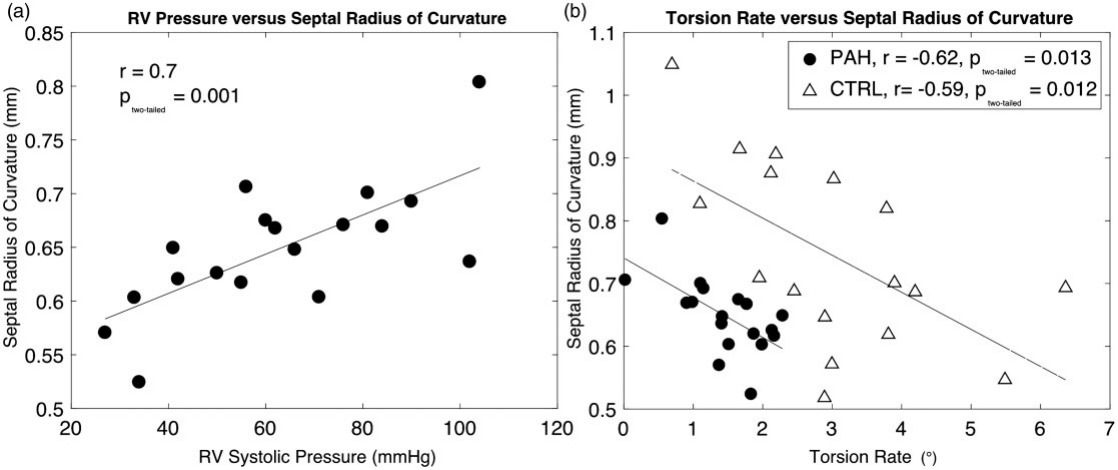
Septal radius of curvature (SROC) is strongly correlated with (a) right ventricular (RV) systolic pressure (*r* = 0.70, *P* = 0.001), and (b) mildly with left ventricular (LV) torsion rate for pulmonary arterial hypertension (PAH) (•, *r* = −0.57, *P* = 0.013), and controls (△, *r* = −0.59, *P* = 0.012).

The SROC in the PAH cohort showed a strong relationship with increasing RV systolic pressure (*P* = 0.001), shown in Fig. 4a, consistent with the mechanism of increasing RV pressure induced septal flattening. SROC further demonstrated a moderate inverse relationship with the LV torsion rate for PAH (*P* = 0.013) and control subjects (Figure 4b) (*P* = 0.012).

### LV torsion rate and catheterization parameters

**Fig. 5.**
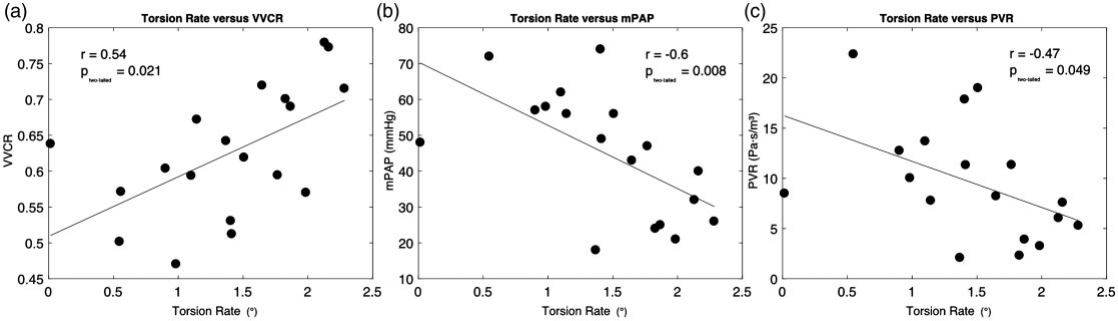
Torsion rates for pulmonary arterial hypertension (PAH) patients are highly correlated with (a) ventricular vascular coupling ratio (VVCR) (*r* = 0.54, *P* = 0.021), (b) mean pulmonary arterial pressure (mPAP) (*r* = −0.60, *P* = 0.008), and (c) pulmonary vascular resistance (PVR) (*r* = −0.47, *P* = 0.049).

VVCR showed a positive relationship with LV torsion rate (Fig. 5a, *P* = 0.021). The torsion rate correlated highly with invasive hemodynamic parameters including mPAP (*P* = 0.008) and PVR (*P* = 0.049), shown in Fig. 5b and c. The torsion rate, however, did not show a significant relationship with arterial compliance (*r* = 0.39, *P* = 0.101).

## Discussion

Utilizing cardiac catheterization and tagged CMR, we analyzed the LV torsion rate in pediatric PAH patients and its relationship to RV function and afterload. Our main findings showed that: (a) LV torsion rate tends to be decreased in PAH subjects compared to controls; and (b) this reduction in torsion rate is concomitant with a reduction in RV contractility. Our results, combined with the current literature on the interventricular mechanical relationship,^8,23–25^ suggests that LV torsion seems to be associated with declining RV function in pediatric PAH. The previous body of research shows a significant reduction in LV strain rate concomitant with functional pulmonary hypertension parameters in pediatric PAH unassociated with left heart disease.^26,27^ However, we are the first directly to relate decreasing LV torsion rate to decreasing RV contractility and VVCR measured by RHC.

### LV torsion rate and RV contractility

A decreasing LV torsion rate shows a relationship with decreasing RV contractility (Fig. 3a). Whether LV torsion is influencing RV contractility or vice versa cannot be definitively determined from our data. However, multiple previous studies show that in healthy subjects, the right ventricle relies on the left ventricle for mechanical support.^8,28,29^ Initial RV dysfunction in PAH is brought on by increasing resistive afterload, thus logically influencing LV twist through IVS alteration. In advanced PAH, where the right ventricle becomes hypertrophied and enlarged, the ability of the right ventricle to affect LV mechanics through this maladaptation may be more significant. Conversely, the subsequent reduction in LV torsion may further drive RV functional decline through reduced assistance from the left ventricle.

### LV torsion rate and RVEF

RVEF has a steeper and stronger correlation with LV torsion rate in the PAH cohort compared to the controls, as seen in Fig. 2b. Therefore, any small changes in LV torsion rate is associated with larger changes in the RVEF in the PAH cohort. Interestingly, the LV torsion rate did not demonstrate a significant relationship with LVEF in either cohort. This suggests that although the LV torsion rate may be reduced in PAH, LV function is preserved while RV function may be declining. We surmise that in PAH children, declining RV function may be highly dependent on declining LV torsion rate, and the right ventricle may have increased sensitivity to changes in LV mechanics, while the left ventricle is able to maintain a normal ejection fraction.

### SROC, RV pressure and VVCR

In our study, a lower LV torsion rate demonstrated a relationship with higher RV systolic pressure (Fig. 3b). Increased RV pressure is known to affect LV mechanics through septal alteration, and a relationship with a reduction in LV strain/strain rate has previously been reported.^30–32^

The hypothesis that the LV torsion rate decreases with increasing RV pressure – in part due to septal flattening – is supported by Fig. 3b. However, the exact causality for this mechanism could be influenced by other factors, and therefore it cannot be stated with complete exclusivity that septal flattening (caused by increased RV pressure) is decreasing LV torsion rate. In addition, SROC demonstrated a strong relationship with RV systolic pressure and LV torsion rate (Fig. 4). The relationship of SROC and the right ventricle demonstrates that, in our cohort, increasing RV pressure affects the geometry of the IVS, and is consistent with previous studies in which SROC has been used as a measurement in the assessment of PAH.^32–35^

VVCR has been shown to have enormous prognostic value for PAH,^36,37^ and demonstrated a direct relationship with the LV torsion rate in this study. VVCR is an indicative metric for when the patient enters a point of maladaptive remodeling and typically experiences a rapid decline. Our finding that the LV torsion rate is highly correlated to both contractility and VVCR implies the potential role it has in PAH related to contractility and afterload (through decreased septal curvature).

### Mechanistic theory

**Fig. 6.**
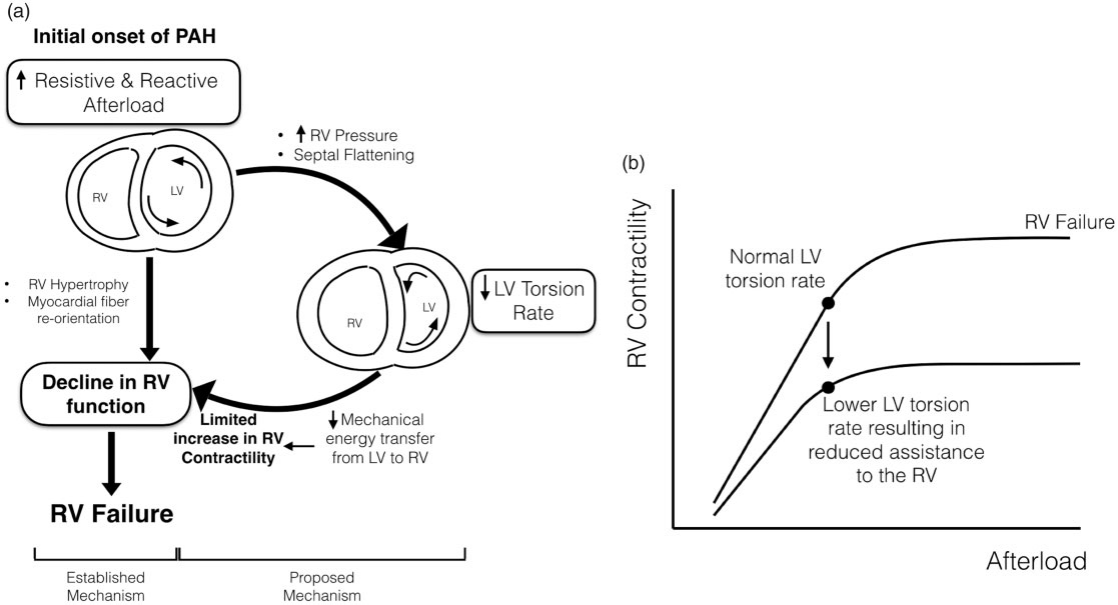
Schematic of proposed pulmonary arterial hypertension (PAH) etiology. (a) The onset of PAH due to increasing resistive and reactive afterload results in a decrease in right ventricular (RV) function due to established mechanisms (left) or results in a reduction in left ventricular (LV) torsion rate, which reduces the amount of mechanical energy transferred from the left to the right ventricle (right). LV torsion rate reduction restricts the increase in RV contractility, eventually resulting in RV functional decline. (b) When LV torsion rate decreases in PAH, RV contractility is reduced, and afterload will outmatch RV contractility, resulting in RV–pulmonary arterial decoupling and RV failure.

We postulate the following mechanism based on the data from this study: increased afterload causes increased RV systolic pressure, which causes a decrease in LV torsion rate which in turn causes further decrease in RV contractility and ventricular decline. Fig. 6a shows our proposed schematic of PAH etiology from mild to severe in which the RV compensatory capability in ventricular–vascular coupling becomes compromised. Based on the assistance that the left ventricle provides the right ventricle through mechanical coupling,^8,28,29^ LV twist may be indicative of the amount of mechanical energy transferred from the left to the right ventricle, and thus reduced LV torsion may result in reduced mechanical energy transfer from the left to the right ventricle during systole (indicated in the right side of Fig. 6a),^9,10,23^ thereby leading to decreased RV contractility. The reduced mechanical energy transferred from the left ventricle results in a decrease in maximum RV functional reserve, allowing the right ventricle eventually to reach a limit where contractility is outmatched by rising afterload. This theory is supported by the relationship of LV torsion with RV contractility (Fig. 3a); however, further research is needed to validate the mechanistic causation. Fig. 6b shows the hypothetical contractility that the right ventricle could generate to overcome afterload with and without the assistance of the left ventricle. Undoubtedly, this multicomponent system is extremely complex, intimately coupled, and progressive in nature. However, the presented data support the proposed mechanisms of biomechanical progression of PAH and warrant additional mechanistic studies.

### Limitations

The implications of our findings are limited by a small number of study subjects, and thus may not fully represent a spectrum of pediatric PAH patients. Control subjects were unable to undergo RHC, therefore a comparative analysis between control and PAH subjects could not be made for all hemodynamic and single-beat derived metrics. The regional and directional strain of the left ventricle was not analyzed in this study; however, future analysis of ventricular torsion will consider strain to analyze regional differences in myocardial motion. The limits of agreement for intraobserver Bland–Altman analysis are approximately within the magnitude of the standard deviation of the LV torsion rate in control subjects, which indicates a limitation in precision of the measurements, and thus the conclusions presented are speculative and warrant additional analysis. Tagged MRI has a limit of detection that is defined as the size of the voxel,^38^ and as such is limited in resolution. In-plane motion is also affected by through-plane motion during ventricular contraction, and this change in through-plane motion was not accounted for. However, a study by Brotman et al. comparing LV rotation calculated with and without through-plane motion demonstrated equal variances between methods, although the methods yielded significantly different values in rotation.^39^ Therefore, within consistent methods of measurement, torsion and rotation can be relatively compared between two populations without the inclusion of through-plane motion. Hence, the measurement of LV rotation and torsion through conventional methods of short-axis image analysis has been widely accepted as a standard method for tissue-tagged CMR studies.^20,40–42^ For future analysis, deformation in the sagittal and coronal planes will be calculated and normalized to short-axis torsion. Although the median age of the PAH cohort^13^ and control subjects^10^ is not significantly different, variance of age within each cohort may influence the differences in LV torsion, as it has been shown that LV torsion increases with age until adulthood.^43,44^

## Conclusion

The LV torsion rate within the pediatric PAH population is reduced compared to normal healthy children and is linearly related to RV contractility and ventricular–vascular coupling. This study suggests that LV mechanics is associated with RV functional decline in pediatric PAH, detectable with non-invasive tagged CMR. We provide a speculative explanation of causality on the underlying physiological mechanism of decreased LV torsion induced reduction of RV contractility. Further mechanistic studies are warranted to elucidate fully the role of LV torsion and its potential as a therapeutic target for improvement of RV function.
